# Supplementary material for: Inferring microevolution from museum collections and resampling: lessons learned from Cepaea
Source: PeerJ. 2017 Oct 27;5:e3938. doi: 10.7717/peerj.3938 (PMC5661451; doi:10.7717/peerj.3938)

Lobith 1960

	n	%
YU (Y00000)	162	51,9
YM (Y00300)	0	0,0
YT (Y00345)	33	22,0
YF (Y12345)	109	72,7
YO (Yellow, other banding categories)	8	5,3
		**100,0**
**Total Ybn (Yellow, banded)**	**150**	48,1
**Total Y (Yellow)**	**312**	84,8
		**100,0**
PU (P00000)	44	78,6
PM (P00300)	0	0,0
PT (P00345)	5	41,7
PF (P12345)	7	58,3
PO (Pink, other banding categories)	0	0,0
		**100,0**
**Total PBn (Pink, banded)**	**12**	21,4
**Total P (Pink)**	**56**	15,2
		**100,0**
BU (B00000)	0	#DEEL/0!
BBn (Brown, banded)	0	#DEEL/0!
		**#DEEL/0!**
**Total B (Brown)**	**0**	0,0
**Total**	**368**	**100,0**
M (*00300)	0	0,0
T (*00345)	38	23,5
F (*12345)	116	71,6
O (other banding categories)	8	4,9
	**162**	**100,0**
YeU (Yellow, effectively unbanded)	199	
other	169	
fused	4	
not fused	79	

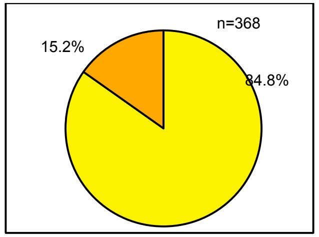

Lobith 1960

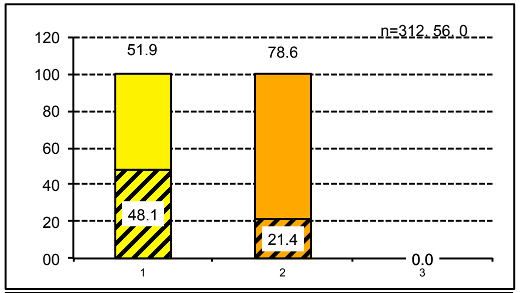

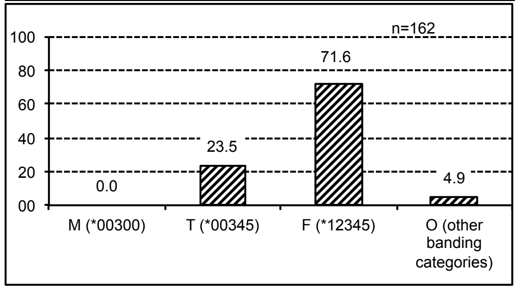

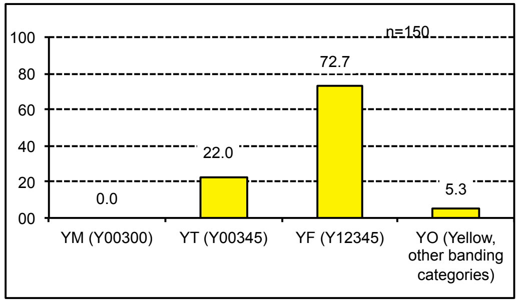

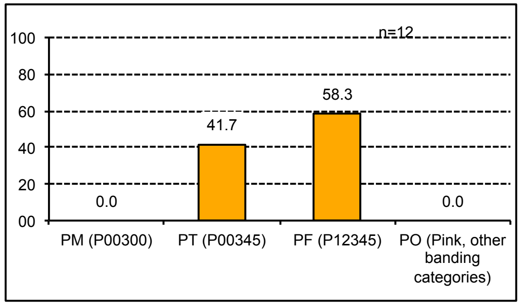

Lobith 1961

	n	%
YU (Y00000)	162	51,6
YM (Y00300)	1	0,7
YT (Y00345)	23	15,1
YF (Y12345)	115	75,7
YO (Yellow, other banding categories)	13	8,6
		**100,0**
**Total Ybn (Yellow, banded)**	**152**	48,4
**Total Y (Yellow)**	**314**	78,9
		**100,0**
PU (P00000)	76	90,5
PM (P00300)	0	0,0
PT (P00345)	2	25,0
PF (P12345)	6	75,0
PO (Pink, other banding categories)	0	0,0
		**100,0**
**Total PBn (Pink, banded)**	**8**	9,5
**Total P (Pink)**	**84**	21,1
		**100,0**
BU (B00000)	0	0,0
BBn (Brown, banded)	0	0,0
		**0,0**
**Total B (Brown)**	**0**	0,0
**Total**	**398**	**100,0**
M (*00300)	1	0,6
T (*00345)	25	15,6
F (*12345)	121	75,6
O (other banding categories)	13	8,1
	**160**	**100,0**
YeU (Yellow, effectively unbanded)	191	
other	207	
fused	3	
not fused	45	

Lobith 1961

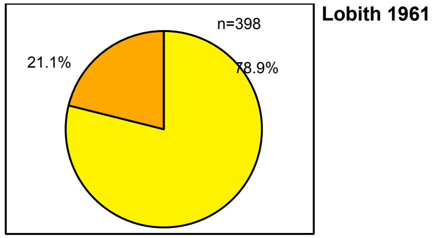

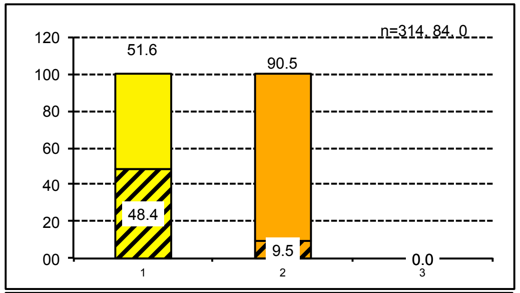

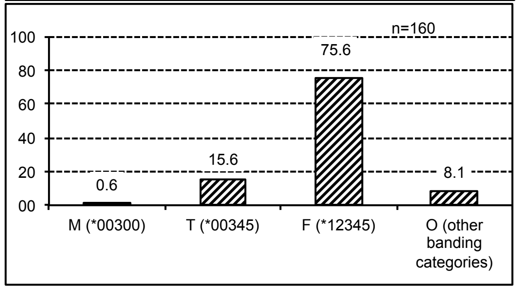

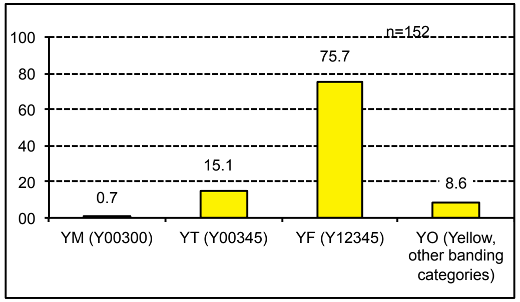

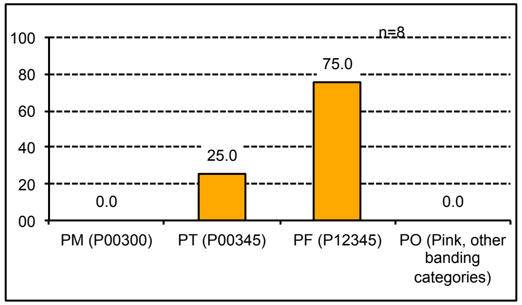

Lobith 1962

	n	%
YU (Y00000)	882	63,7
YM (Y00300)	3	0,6
YT (Y00345)	106	21,1
YF (Y12345)	364	72,5
YO (Yellow, other banding categories)	29	5,8
		**100,0**
**Total Ybn (Yellow, banded)**	**502**	36,3
**Total Y (Yellow)**	**1384**	83,5
		**100,0**
PU (P00000)	218	79,9
PM (P00300)	0	0,0
PT (P00345)	9	16,4
PF (P12345)	44	80,0
PO (Pink, other banding categories)	2	3,6
		**100,0**
**Total PBn (Pink, banded)**	**55**	20,1
**Total P (Pink)**	**273**	16,5
		**100,0**
BU (B00000)	0	0,0
BBn (Brown, banded)	0	0,0
		**0,0**
**Total B (Brown)**	**0**	0,0
**Total**	**1657**	**100,0**
M (*00300)	3	0,5
T (*00345)	115	20,6
F (*12345)	408	73,2
O (other banding categories)	31	5,6
	**557**	**100,0**
YeU (Yellow, effectively unbanded)	1012	
other	645	
fused	9	
not fused	203	

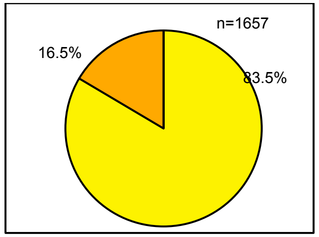

Lobith 1962

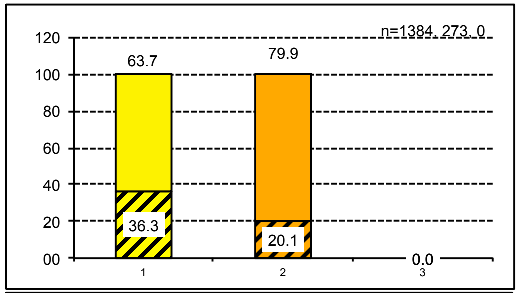

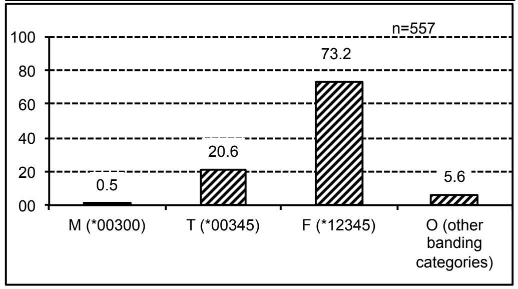

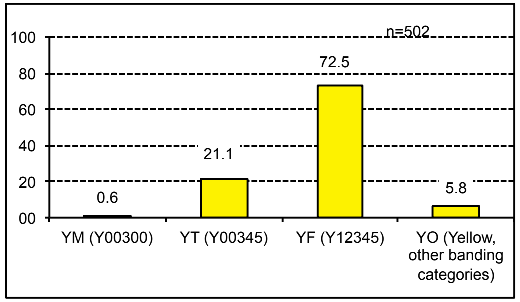

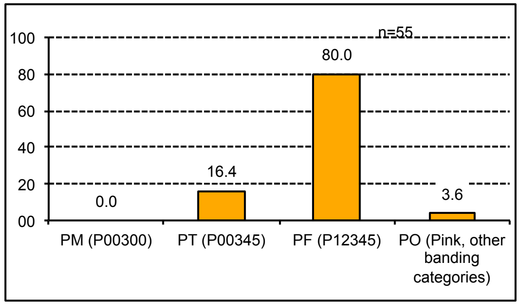

Lobith 2010

	n	%
YU (Y00000)	63	57,8
YM (Y00300)	0	0,0
YT (Y00345)	8	17,4
YF (Y12345)	25	54,3
YO (Yellow, other banding categories)	13	28,3
		**100,0**
**Total Ybn (Yellow, banded)**	**46**	42,2
**Total Y (Yellow)**	**109**	83,2
		**100,0**
PU (P00000)	15	75,0
PM (P00300)	0	0,0
PT (P00345)	2	40,0
PF (P12345)	3	60,0
PO (Pink, other banding categories)	0	0,0
		**100,0**
**Total PBn (Pink, banded)**	**5**	25,0
**Total P (Pink)**	**20**	15,3
		**100,0**
BU (B00000)	2	100,0
BBn (Brown, banded)	0	0,0
		**100,0**
**Total B (Brown)**	**2**	1,5
**Total**	**131**	**100,0**
M (*00300)	0	0,0
T (*00345)	10	19,6
F (*12345)	28	54,9
O (other banding categories)	13	25,5
	**51**	**100,0**
YeU (Yellow, effectively unbanded)	83	
other	48	
fused	1	
not fused	24	

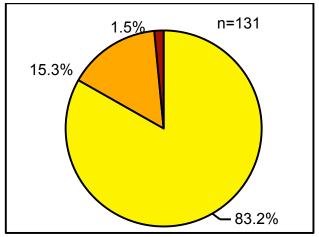

Lobith 2010

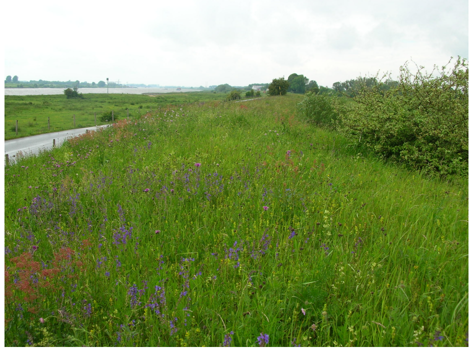

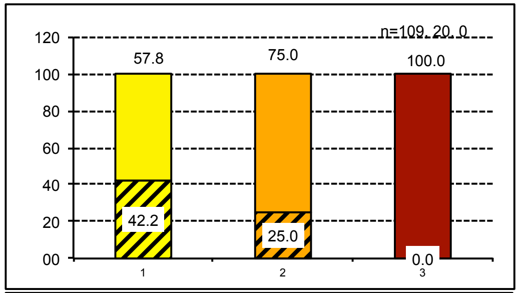

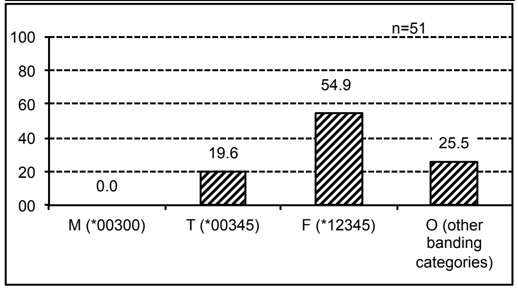

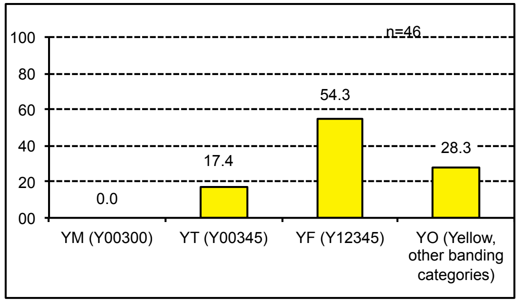

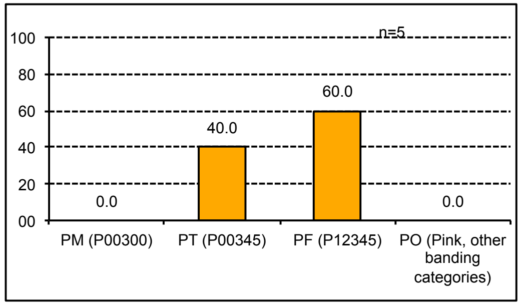

Supplement: Data S2 — Full details of the original and the resampled collections from Lobith, including a photo of the contemporary habitat. [file peerj-05-3938-s002.pdf]
