# Supplementary material for: Inferring microevolution from museum collections and resampling: lessons learned from Cepaea
Source: PeerJ. 2017 Oct 27;5:e3938. doi: 10.7717/peerj.3938 (PMC5661451; doi:10.7717/peerj.3938)

Empe 1915

	n	%
YU (Y00000)	151	43,1
YM (Y00300)	0	0,0
YT (Y00345)	151	75,9
YF (Y12345)	47	23,6
YO (Yellow, other banding categories)	1	0,5
		**100,0**
**Total Ybn (Yellow, banded)**	**199**	56,9
**Total Y (Yellow)**	**350**	74,8
		**100,0**
PU (P00000)	45	43,3
PM (P00300)	0	0,0
PT (P00345)	37	62,7
PF (P12345)	22	37,3
PO (Pink, other banding categories)	0	0,0
		**100,0**
**Total PBn (Pink, banded)**	**59**	56,7
**Total P (Pink)**	**104**	22,2
		**100,0**
BU (B00000)	14	100,0
BBn (Brown, banded)	0	0,0
		**100,0**
**Total B (Brown)**	**14**	3,0
**Total**	**468**	**100,0**
M (*00300)	0	0,0
T (*00345)	188	72,9
F (*12345)	69	26,7
O (other banding categories)	1	0,4
	**258**	**100,0**
YeU (Yellow, effectively unbanded)	303	
other	165	
fused	13	
not fused	55	

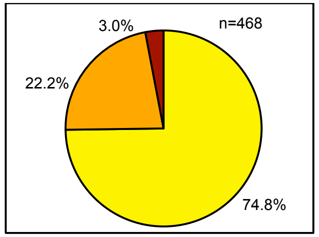

Empe 1915

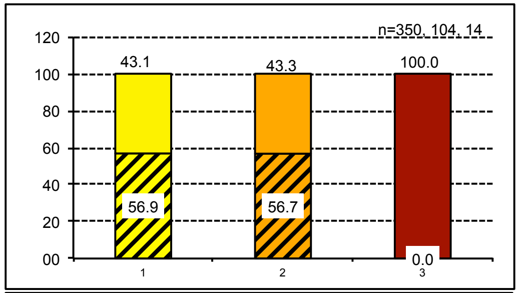

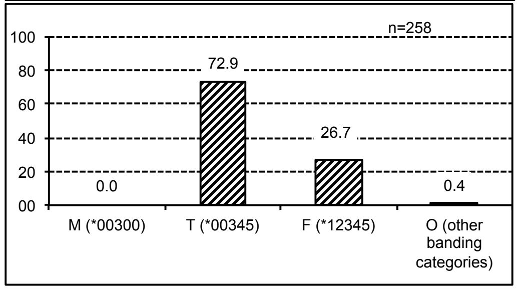

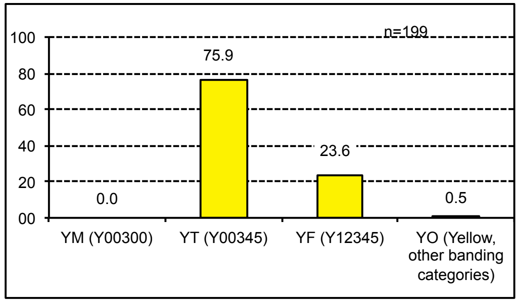

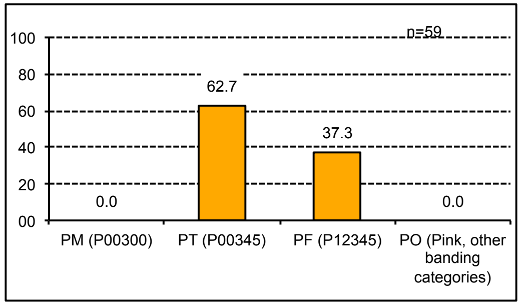

Empe 1951

	n	%
YU (Y00000)	112	49,8
YM (Y00300)	0	0,0
YT (Y00345)	86	76,1
YF (Y12345)	27	23,9
YO (Yellow, other banding categories)	0	0,0
		**100,0**
**Total Ybn (Yellow, banded)**	**113**	50,2
**Total Y (Yellow)**	**225**	66,2
		**100,0**
PU (P00000)	62	53,9
PM (P00300)	0	0,0
PT (P00345)	41	77,4
PF (P12345)	12	22,6
PO (Pink, other banding categories)	0	0,0
		**100,0**
**Total PBn (Pink, banded)**	**53**	46,1
**Total P (Pink)**	**115**	33,8
		**100,0**
BU (B00000)	0	0,0
BBn (Brown, banded)	0	0,0
		**0,0**
**Total B (Brown)**	**0**	0,0
**Total**	**340**	**100,0**
M (*00300)	0	0,0
T (*00345)	127	76,5
F (*12345)	39	23,5
O (other banding categories)	0	0,0
	**166**	**100,0**
YeU (Yellow, effectively unbanded)	198	
other	142	
fused	7	
not fused	32	

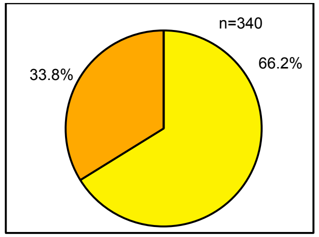

Empe 1951

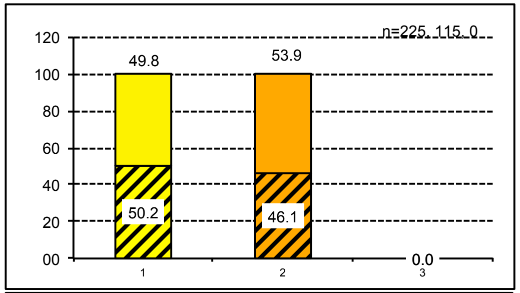

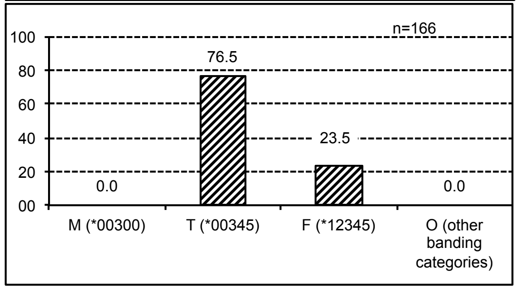

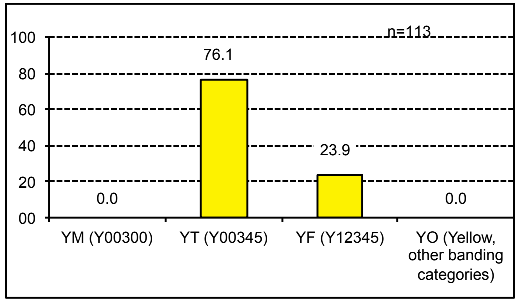

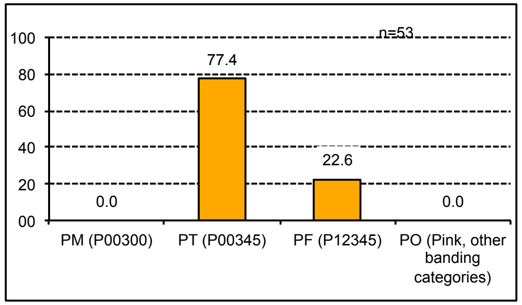

Empe 2010 reeds

	n	%
YU (Y00000)	21	28,8
YM (Y00300)	1	1,9
YT (Y00345)	34	65,4
YF (Y12345)	17	32,7
YO (Yellow, other banding categories)	0	0,0
		**100,0**
**Total Ybn (Yellow, banded)**	**52**	71,2
**Total Y (Yellow)**	**73**	77,7
		**100,0**
PU (P00000)	12	63,2
PM (P00300)	0	0,0
PT (P00345)	2	28,6
PF (P12345)	5	71,4
PO (Pink, other banding categories)	0	0,0
		**100,0**
**Total PBn (Pink, banded)**	**7**	36,8
**Total P (Pink)**	**19**	20,2
		**100,0**
BU (B00000)	2	100,0
BBn (Brown, banded)	0	0,0
		**100,0**
**Total B (Brown)**	**2**	2,1
**Total**	**94**	**100,0**
M (*00300)	1	1,7
T (*00345)	36	61,0
F (*12345)	22	37,3
O (other banding categories)	0	0,0
	**59**	**100,0**

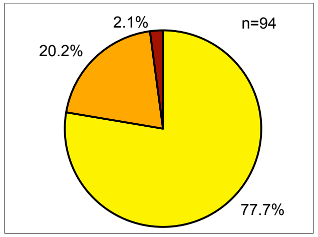

Empe 2010 (reeds)

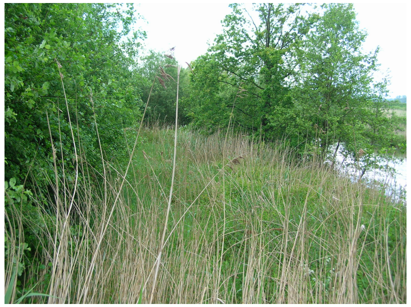

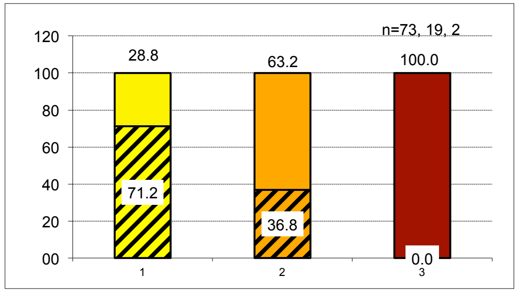

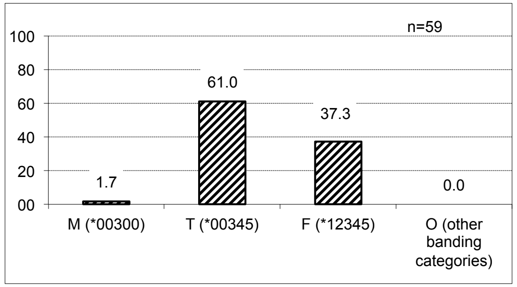

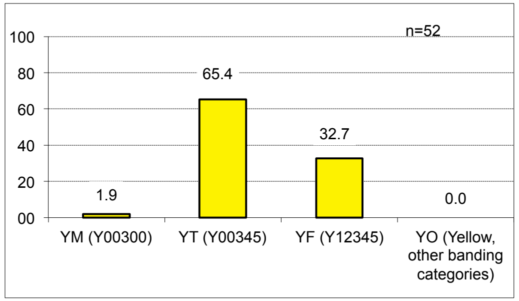

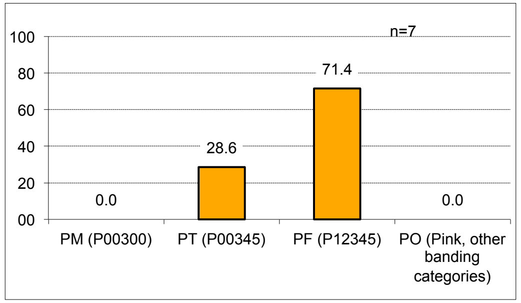

Empe 2010 S

	n	%
YU (Y00000)	24	33,3
YM (Y00300)	1	2,1
YT (Y00345)	34	70,8
YF (Y12345)	13	27,1
YO (Yellow, other banding categories)	0	0,0
		**100,0**
**Total Ybn (Yellow, banded)**	**48**	66,7
**Total Y (Yellow)**	**72**	75,8
		**100,0**
PU (P00000)	15	65,2
PM (P00300)	0	0,0
PT (P00345)	7	87,5
PF (P12345)	1	12,5
PO (Pink, other banding categories)	0	0,0
		**100,0**
**Total PBn (Pink, banded)**	**8**	34,8
**Total P (Pink)**	**23**	24,2
		**100,0**
BU (B00000)	0	0,0
BBn (Brown, banded)	0	0,0
		**0,0**
**Total B (Brown)**	**0**	0,0
**Total**	**95**	**100,0**
M (*00300)	1	1,8
T (*00345)	41	73,2
F (*12345)	14	25,0
O (other banding categories)	0	0,0
	**56**	**100,0**

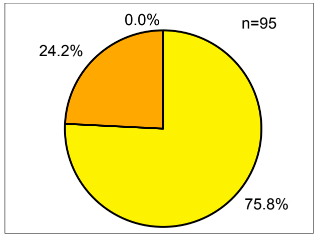

Empe 2010 S
S wood

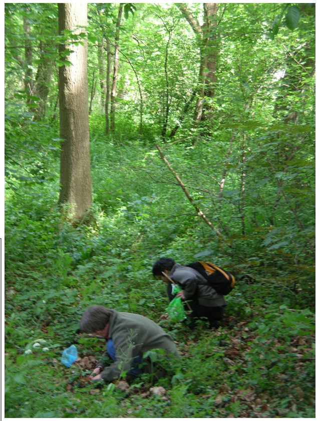

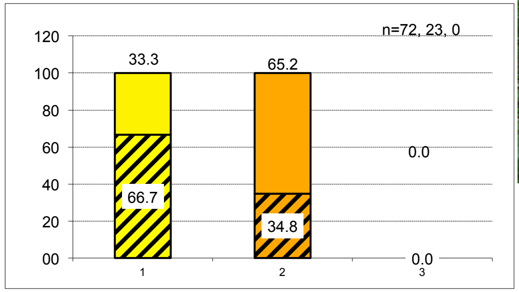

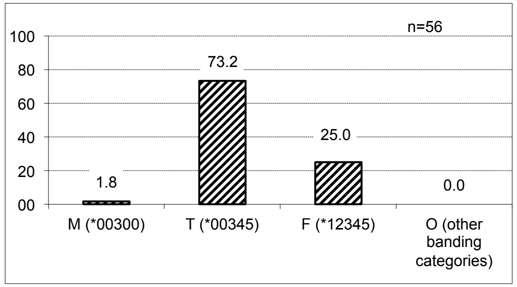

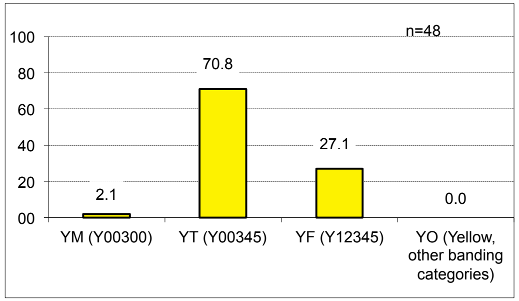

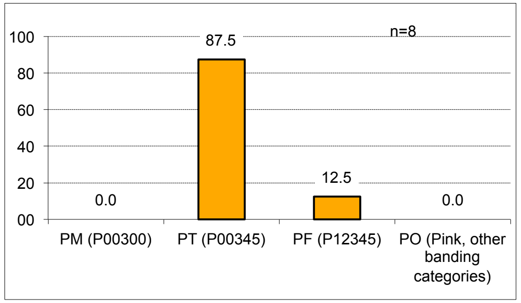

Empe 2010 pooled

	n	%
YU (Y00000)	45	31,0
YM (Y00300)	2	2,0
YT (Y00345)	68	68,0
YF (Y12345)	30	30,0
YO (Yellow, other banding categories)	0	0,0
		**100,0**
**Total Ybn (Yellow, banded)**	**100**	69,0
**Total Y (Yellow)**	**145**	76,7
		**100,0**
PU (P00000)	27	64,3
PM (P00300)	0	0,0
PT (P00345)	9	60,0
PF (P12345)	6	40,0
PO (Pink, other banding categories)	0	0,0
		**100,0**
**Total PBn (Pink, banded)**	**15**	35,7
**Total P (Pink)**	**42**	22,2
		**100,0**
BU (B00000)	2	100,0
BBn (Brown, banded)	0	0,0
		**100,0**
**Total B (Brown)**	**2**	1,1
**Total**	**189**	**100,0**
M (*00300)	2	1,7
T (*00345)	77	67,0
F (*12345)	36	31,3
O (other banding categories)	0	0,0
	**115**	**100,0**
YeU (Yellow, effectively unbanded)	115	
other	74	
fused	2	
not fused	28	

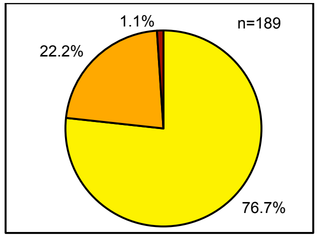

Empe 2010 (pooled)

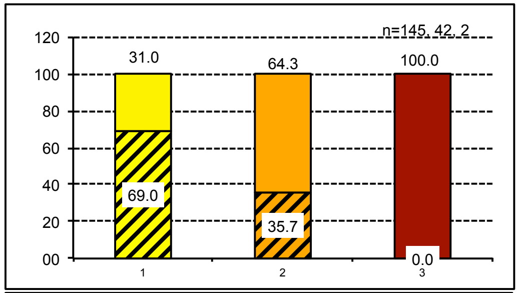

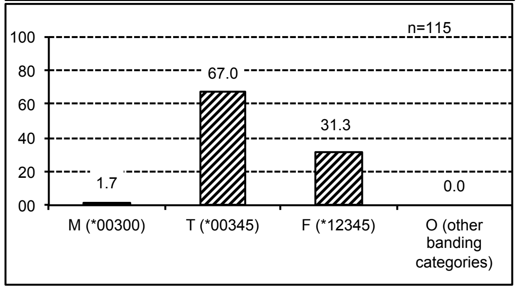

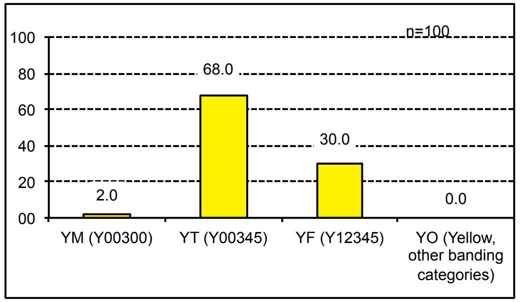

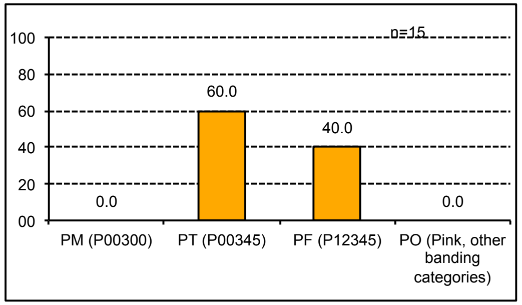

Supplement: Data S3 — Full details of the original and the resampled collections from Empe, including a photo of the contemporary habitats. [file peerj-05-3938-s003.pdf]
