# Supplementary material for: Inferring microevolution from museum collections and resampling: lessons learned from Cepaea
Source: PeerJ. 2017 Oct 27;5:e3938. doi: 10.7717/peerj.3938 (PMC5661451; doi:10.7717/peerj.3938)

Allemansgeest 1942

	n	%
YU (Y00000)	8	3,0
YM (Y00300)	84	32,1
YT (Y00345)	53	20,2
YF (Y12345)	123	46,9
YO (Yellow, other banding categories)	2	0,8
		**100,0**
**Total Ybn (Yellow, banded)**	**262**	97,0
**Total Y (Yellow)**	**270**	54,0
		**100,0**
PU (P00000)	162	70,4
PM (P00300)	9	13,2
PT (P00345)	14	20,6
PF (P12345)	45	66,2
PO (Pink, other banding categories)	0	0,0
		**100,0**
**Total PBn (Pink, banded)**	**68**	29,6
**Total P (Pink)**	**230**	46,0
		**100,0**
BU (B00000)	0	0,0
BBn (Brown, banded)	0	0,0
		**0,0**
**Total B (Brown)**	**0**	0,0
**Total**	**500**	**100,0**
M (*00300)	93	28,2
T (*00345)	67	20,3
F (*12345)	168	50,9
O (other banding categories)	2	0,6
	**330**	**100,0**
YeU (Yellow, effectively unbanded)	146	
other	354	

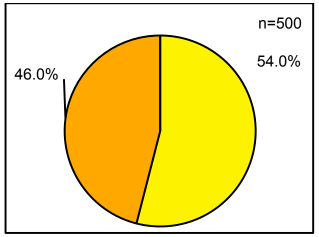

Allemansgeest 1942

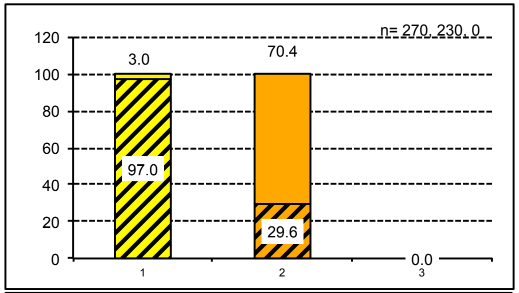

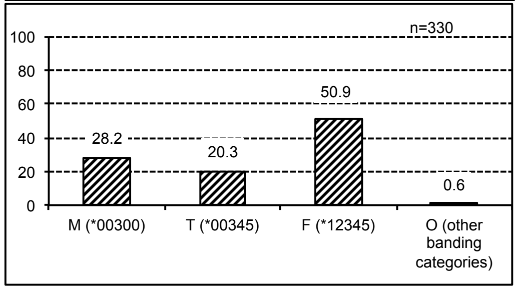

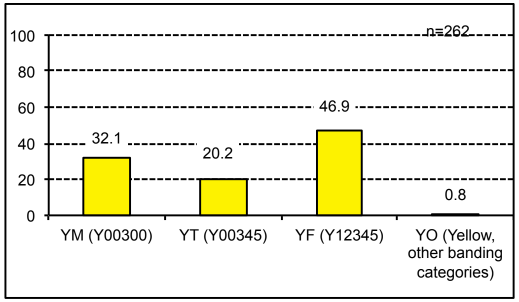

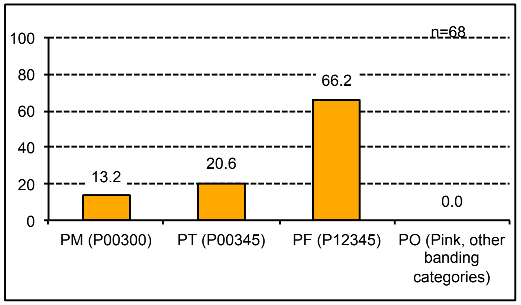

Allemansgeest 1943

	n	%
YU (Y00000)	11	2,7
YM (Y00300)	187	47,6
YT (Y00345)	94	23,9
YF (Y12345)	102	26,0
YO (Yellow, other banding categories)	10	2,5
		**100,0**
**Total Ybn (Yellow, banded)**	**393**	97,3
**Total Y (Yellow)**	**404**	52,3
		**100,0**
PU (P00000)	235	63,9
PM (P00300)	68	51,1
PT (P00345)	36	27,1
PF (P12345)	27	20,3
PO (Pink, other banding categories)	2	1,5
		**100,0**
**Total PBn (Pink, banded)**	**133**	36,1
**Total P (Pink)**	**368**	47,7
		**100,0**
BU (B00000)	0	0,0
BBn (Brown, banded)	0	0,0
		**0,0**
**Total B (Brown)**	**0**	0,0
**Total**	**772**	**100,0**
M (*00300)	255	48,5
T (*00345)	130	24,7
F (*12345)	129	24,5
O (other banding categories)	12	2,3
	**526**	**100,0**
YeU (Yellow, effectively unbanded)	298	
other	474	

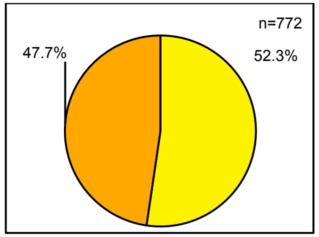

Allemansgeest 1943

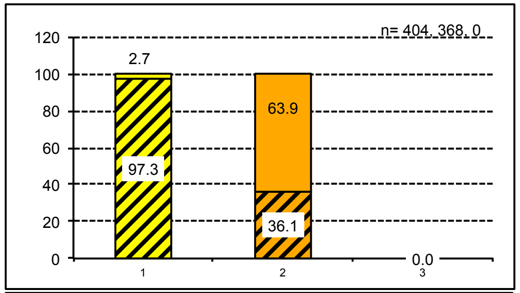

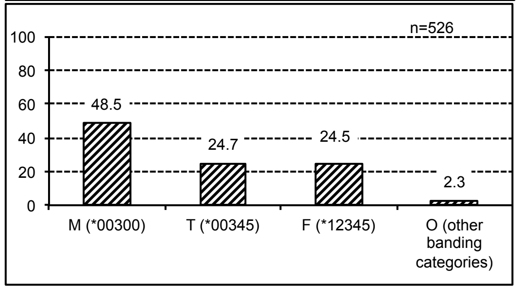

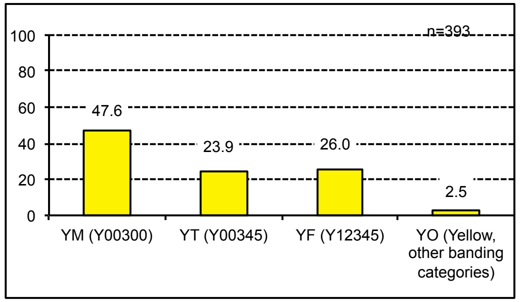

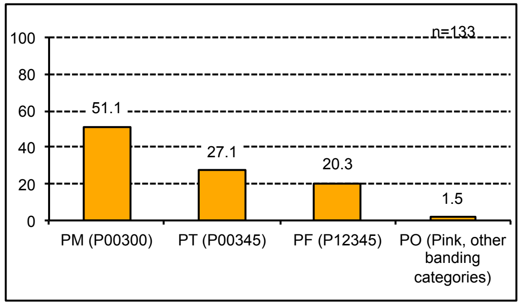

Allemansgeest 2010

	n	%
YU (Y00000)	19	39,6
YM (Y00300)	10	34,5
YT (Y00345)	9	31,0
YF (Y12345)	8	27,6
YO (Yellow, other banding categories)	2	6,9
		**100,0**
**Total Ybn (Yellow, banded)**	**29**	60,4
**Total Y (Yellow)**	**48**	62,3
		**100,0**
PU (P00000)	21	72,4
PM (P00300)	4	50,0
PT (P00345)	2	25,0
PF (P12345)	2	25,0
PO (Pink, other banding categories)	0	0,0
		**100,0**
**Total PBn (Pink, banded)**	**8**	27,6
**Total P (Pink)**	**29**	37,7
		**100,0**
BU (B00000)	0	0,0
BBn (Brown, banded)	0	0,0
		**0,0**
**Total B (Brown)**	**0**	0,0
**Total**	**77**	**100,0**
M (*00300)	14	37,8
T (*00345)	11	29,7
F (*12345)	10	27,0
O (other banding categories)	2	5,4
	**37**	**100,0**
YeU (Yellow, effectively unbanded)	38	
other	39	

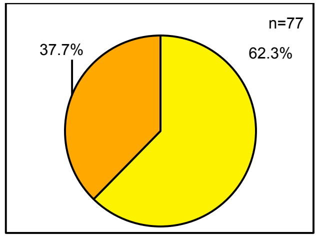

Allemansgeest 2010

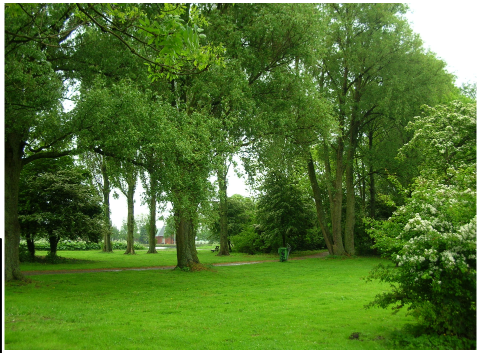

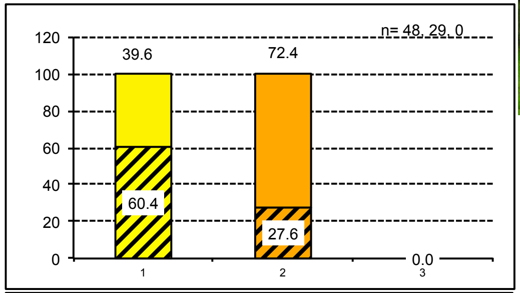

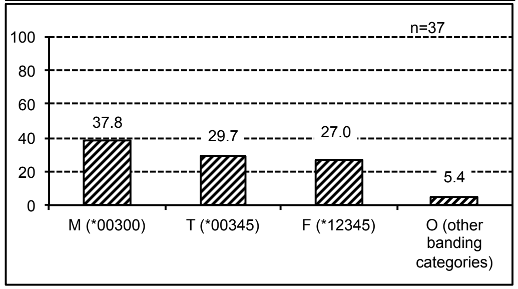

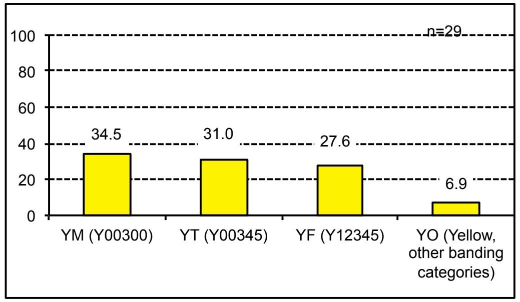

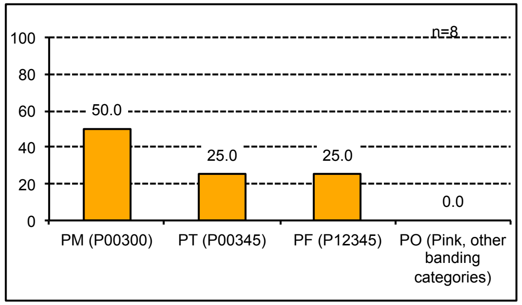

Supplement: Data S4 — Full details of the original and the resampled collections from Allemansgeest, including a photo of the contemporary habitat. [file peerj-05-3938-s004.pdf]
